# Nutritional value of school meals and their contributions to energy and nutrient intakes of rural school children in Enugu and Anambra States, Nigeria

**DOI:** 10.1186/s40795-018-0216-0

**Published:** 2018-02-21

**Authors:** Rufina N. B. Ayogu, Paul E. Eme, Vivien C. Anyaegbu, Henrietta N. Ene-Obong, Uche V. Amazigo

**Affiliations:** 1 Department of Home Science, Nutrition and Dietetics, University of Nigeria, Nsukka, Nigeria; 2 Department of Biochemistry (Human Nutrition and Dietetics Unit), University of Calabar, Calabar, Nigeria; 3 Pan African Community Initiative on Education and Health (PACIEH), Enugu, Canada

**Keywords:** Contributions, School meals, RNI, Energy and nutrient intakes, Rural school children, Enugu state, Anambra state, Nigeria

## Abstract

**Background:**

Nutritional deficiencies among school children may hinge on inadequate nutrient intake. School meals should improve nutrient intakes by providing a third of recommended daily energy and nutrient intakes (RNI). The study aimed at evaluating school meals served in three rural schools to determine if they met one third of the RNI of the children. This will enhance meal planning.

**Methods:**

Food samples (20 g) that constituted the school meals were collected for five consecutive days from three schools where school lunch programme was implemented. These were put in labelled small air tight plastic containers and stored in deep freezers in the Department of Home Science, Nutrition and Dietetics, University of Nigeria, Nsukka. The samples were analysed chemically using standard methods. Portion sizes of foods were obtained and the contributions made by these meals to the children’s RNI were calculated. Results were presented in percentages and means ± standard deviations.

**Results:**

The results showed that energy value of the meals ranged from 32.27 – 243.4 Kcal/100 g. The school meals contained carbohydrate (0.7 – 48.4 g), protein (0.69 – 12.6 g), vitamin C (0.7 – 8.22 mg), vitamin A (3.0 – 255.5 RE), iron (0.05 – 1.7 mg), calcium (3.0 –120 mg) and zinc (0.14 – 3.0 mg) per 100 g of food consumed. They contributed 16.4 – 25.5% energy, 53.4 – 116.9% protein, 66.0 – 159.5% vitamin A, 37.3 – 45.7% vitamin C, 13.2 – 28.5% calcium, 5.9 – 20.6% iron and 35.1 – 92.9% zinc to the children’s daily requirements.

**Conclusion:**

The school meals provided over one third of the RNI for protein, vitamins A and C, and zinc but did not meet a third of the RNI for energy, calcium and iron.

## Background

The high prevalence of under nutrition in developing countries of the world has triggered many intervention strategies to ameliorate these conditions which have been found to have huge consequences on the affected individuals, their families and the nation as a whole. One of such strategies with focus on school children is school health programme which incorporates school meal/lunch. School meal programme ensures that the school child receives one nutritious mid day meal every school day to grow and develop adequately. The ubiquity of school feeding programmes suggests that these programmes are indeed appropriate candidates for a rapid safety net response [1]. Household meals in most low- and middle-income countries have been observed to be inadequate in energy, animal protein and micronutrients. School meals therefore complement what school children in these homes receive. Bundy et al. [1] explained that there are very few studies that compare in-school feeding with take-home rations in similar settings, and the few that have gone further with this suggest that both programmes lead to similar improvements over having no programme at all.

School-aged children require adequate food in both quantity and quality to stay healthy. Provision of energy, immunity, regulation of body processes, repair and maintenance of body tissues can only be achieved through adequate food intake. Snacks consumed in-between meals also add to total food intake necessary to meet the recommended daily energy and nutrient intakes of school children. From a health promotion approach, the quality and amount of food and beverages consumed while at school have an enormous potential impact on the health and well-being of young people [2]. They are active and often require more calories and nutrients in comparison with their body sizes.

Breakfast skipping is common among school children [3, 4] for many reasons such as lack of food and time to consume what is available [4]. School period lasts 6 – 8 h daily and the children are expected to endure hunger throughout this period. When hungry, one finds it difficult to cope with school activities hence the need for school meals. School meals alleviate short term hunger, and provide energy and nutrients for growth and development of the school aged child. Adequate nutrition guarantees better health status and ensures sustained attention span for better academic performances. Access to a nutritious breakfast and mid-day meal is a very important determinant of the nutritional status as well as the overall well-being and cognitive development of school children. Healthier and better nourished children stay in school longer, learn more and later become healthier and more productive adults [5].

Enugu State is one of the 13 states where the Federal Government of Nigeria implemented school feeding programme in 2004 using locally grown foods. With the collapse of the programme in the State, school children in the State were further exposed to nutritional challenges. Among 5 – 12-year-old school children in Enugu State, Ejekwu et al. [6] reported that 27.7% were stunted, 29.9% were underweight and 25.5% were wasted and showed that rural children were significantly (*P < 0.05*) more stunted than those in the urban areas. Nwamara et al. [7] also reported stunting (19.5%), wasting (8.9%), underweight (8.5%) and iodine deficiency (3.8%) among primary school children in Okpuje, Nsukka Local Government Area (LGA) of Enugu State. In Anambra State, prevalence of underweight and stunting among primary school pupils were reported as 10.7 and 1.9%, respectively [8]. Ukegbu (4) reported that 15.9, 27.4 and 20.7% of primary school children in Orumba North LGA of Anambra State were underweight, stunted and wasted, respectively. Indicators of anthropometric and micronutrient status among school children in various parts of Nigeria [9–14] indicate that school children’s nutritional status is substandard and unsatisfactory.

In the light of this, a non – governmental, not-for-profit organisation, Pan African Community Initiative on Education and Health (PACIEH) adopted a community-managed strategy [15] to improve the health, nutrition and education of school children in three rural schools in Enugu and Anambra States of Nigeria. It currently feeds 719 pupils every school day with funds provided by Heineken African Foundation (through Nigeria Breweries PLC), PACIEH, Enugu State Universal Basic Education Board (ENSUBEB) and TruValue). The menu was altered a few times to what is shown in this study to improve nutrient intakes while contending with frequent increase in the cost of food items. The change of menu necessitated this study aimed at evaluating the nutritional value of the meals consumed by the school children and determining the contributions they made to the recommended daily energy and nutrient intakes of the children. This would enhance review of the school menu for better energy and nutrient intakes. It would also inform policy makers on school meal planning.

## Methods

### Study area

The study took place in June, 2015 at Eke Central Primary School and Oma-Eke Community Primary School both in Udi LGA of Enugu State and at Central/Salvation Primary School Afor-agu, Abatete in Idemili North LGA of Anambra State, Nigeria. These are remote resource poor communities. The parents of the pupils and the School Based Management Committee (SBMC) played a major role in determining the menu at each school. This is based on the foods available in the community. Each of the three schools has school kitchen (built by PACIEH and partners) where the foods were cooked centrally by trained volunteer female cooks who were parents of the school children. Primary training of the cooks took place before the commencement of the feeding programme in 2013 and subsequent retraining was given at the beginning of each school year. The training involved both theory and practical demonstrations on measurements of food items, food preparation, portion sizes and personal, environmental and food hygiene practices. The cooks also shared the foods to the children in their classrooms between 10.30 and 11.30 am every school day. The meals were common to all the children.

### Ethics approval and consent to participate

A meeting was held with the School Based Management Committee (SBMC) and Parents Teachers’ Association (PTA) of the schools and the objective of the study explained to them and their consent obtained. The children gave oral consent for their foods to be weighed. PACIEH and partners (Enugu State Universal Basic Education Board (ENSUBEB) and Ministry of Education) approved the study.

### Collection of food samples

Samples (20 g) of each cooked food were collected individually from each of the three primary schools on 5 consecutive schools days. Bread, yogurt, milk, eggs and pap were not collected for analysis; their values in the West African Food Composition Table (FCT) [16] were used for computation. The samples were preserved in labelled small air tight plastic containers and stored in a deep freeze (-20 °C) in the Diet Therapy Laboratory of the Department of Home Science, Nutrition and Dietetics, University of Nigeria, Nsukka until analysis.

### Chemical analyses of the school meal

Triplicate determinations of proximate and micronutrient compositions of the school meals were carried out at the analytical laboratory of the Department of Home Science, Nutrition and Dietetics by the methods described by Association of Analytical Chemists (AOAC) [17]. Micro-Kjeldahl method was used for protein, dry ashing for ash, soxhlet extraction method for fat, hot air oven method for moisture, acid hydrolysis for crude fibre and carbohydrate was determined by difference. Atomic absorption Sphectrophotometric method was employed for mineral (iron, zinc and calcium) and vitamin (vitamin A and C) determinations. The nutrient values of foods not analyzed were obtained from West African Food Composition Table (FCT) [16] and used in calculating the contributions to energy and nutrient intakes made by such foods. Atwater conversion factor [18] was used in the determination of the energy values of the foods.

### Determination of portion sizes of the meals consumed by the pupils

Portion sizes refer to the quantity of food consumed. Determination of the portion sizes was carried out for every school child (719) in each of the schools on all 5 school days of the week. These were presented according to the foods consumed by the school children.

Four stages were involved:

i. An empty plate was weighed for each school child.
ii. Food was added to the plate and the weight recorded.
iii. The children were given the food to eat and after eating, any leftover in the plate as well as any waste were weighed.
iv. The weight of the leftover food and wastes were subtracted from the quantity served each child to obtain the actual quantity of food consumed (food intake).

### Determination of nutrient intake

This was determined by simple proportion. Results of nutrient analysis carried out by laboratory analysis or obtained from FCT per 100 g and portion sizes in grams were used to obtain the value of nutrients consumed over a period of five school days. For example, if 100 g of steamed bambara groundnut pudding contained X g of protein and the portion size consumed was 200 g, then the protein value consumed $=\left(\frac{X}{100}\times \frac{200}{1}\right)g$ protein.

This was done for all the nutrients involved in the study and the mean intake supplied by the school meal was obtained by dividing the values by 5 (days).

### Statistical analysis

Data generated from the study were analysed using Statistical Package for Social Sciences (SPSS) version 16. Descriptive statistics were used and results were presented in percentages, means and standard deviations. Analysis of variance was used in data analysis and Duncan’s New Multiple Range test was used to separate and compare the means with significance set at *P < 0.05*.

## Results

### Characteristics of the subjects and the school meals

**Fig. 1:**
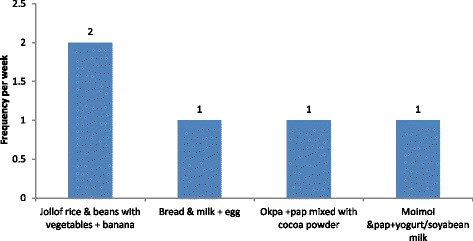
Types and frequency of consumption of school meals in a week

The school children were aged 2 – 5 years (33.0%), 6 – 9 years (34.1%), 10 – 14 years (29.1%), and 15 – 18 years (3.8%). Boys constituted 52.6% while 47.4% were girls. Those in Early Child Centres (ECC) were 36.2% whereas 63.8% were in primary schools. The school meal consisted of jollof rice and beans with vegetables and a finger of banana (consumed twice weekly); steamed cowpea pudding (*moi-moi*) consumed with yogurt and pap (fermented maize paste) mixed with cocoa (richoco) powder; steamed bambara groundnut pudding with pap (fermented maize paste) mixed with cocoa (richoco) powder; and bread, egg with cow milk (Fig. 1). School children at Abatete School did not consume yogurt but soya bean milk.

### Portion sizes of foods consumed

Table 1 shows the mean portion sizes of the foods consumed by the children by age and schools. In all the 3 schools, the portion sizes consumed by the 2 – 5-year-olds were the smallest and significantly (*P < 0.05*) differed from the portion sizes of the other 3 older age groups. The portion sizes of most foods consumed by the 10 – 14 and 15 – 18-year-olds in all the schools were comparable (*P > 0.05).* The quantity of pap mixed with cocoa powder and steamed bambara groundnut pudding *(okpa)* ranged from 170 to 220 g with the 10 – 14 and 15 – 18 years having significantly (*P < 0.05*) higher quantities. The quantity of steamed cowpea pudding *(moi-moi)* consumed by pupils ranged from 170 to 230 g; those aged 15 – 18 years had significantly (*P < 0.05*) higher portion sizes than the other age groups. The amount of bread consumed by the children ranged from 65 to 85 g with pupils of 6 – 9 years at Eke Central Primary School having a significantly (*P < 0.05*) higher quantity than others. The weight of egg consumed by the children ranged from 70 to 85 g and jollof rice and beans with banana ranged from 210 to 440 g; the 15 – 18-year-olds at Oma-Eke Community Primary School and Abatete Primary School had significantly (*P < 0.05*) larger portion sizes in comparison with other age groups. The quantity of cow milk and soya bean milk consumed by the pupils ranged from 180 to 240 ml, respectively; children (15 – 18 years) at Abatete had the highest quantity (*P < 0.05*) of both. Yogurt ranged from 180 to 220 ml; the 15 – 18-year-old pupils at Eke Central Primary School had the largest portion size (*P < 0.05*).

**Table 1:** Portion sizes of school meals consumed by the school children by age and schools

	Age groups			
Variables	2 – 5 years (*N* = 231)	6 – 9 years (*N* = 239)	10 – 14 years (*N* = 204)	15 – 18 years (*N* = 26)
Eke Central Primary School (*N* = 324)	*N* = 106	*N* = 86	*N* = 130	N = 2
*Pap* + *richoco* (g)	175^a^ ± 1.22	195^b^ ± 0.99	220^c^ ± 0.88	220^c^ ± 0.46
*Okpa* (g)	170^a^ ± 2.46	190^b^ ± 1.99	220^c^ ± 1.06	220^c^ ± 0.96
Bread (g)	65^a^ ± 0.79	85^c^ ± 0.71	80^b^ ± 0.86	80^b^ ± 0.61
Egg (g)	70^a^ ± 1.73	70^a^ ± 1.43	80^b^ ± 0.20	80^b^ ± 0.18
Milk (ml)	190^a^ ± 0.57	220^b^ ± 0.51	220^b^ ± 0.49	220^b^ ± 0.40
*Moi-moi* (g)	170^a^ ± 2.36	210^b^ ± 2.08	220^c^ ± 1.96	230^d^ ± 1.89
Yoghurt (ml)	180^a^ ± 0.91	190^b^ ± 0.89	210^c^ ± 1.85	220^d^ ± 1.81
^c^Rice & beans jollof+vegetables (g)	240^a^ ± 0.33	280^b^ ± 0.33	350^d^ ± 1.29	340^c^ ± 0.22
Oma-Eke CPS (*N* = 170)	*N* = 41	*N* = 78	*N* = 50	N = 1
*Pap* + *richoco* (g)	170^a^ ± 2.12	200^b^ ± 0.99	220^d^ ± 1.07	210^c^ ± 0.98
*Okpa* (g)	170^a^ ± 0.32	220^b^ ± 0.33	220^b^ ± 0.42	220^b^ ± 0.30
Bread (g)	70^a^ ± 3.31	75^ab^ ± 2.98	80^b^ ± 2.07	80^b^ ± 1.99
Egg (g)	70^a^ ± 4.38	70^a^ ± 4.02	75^ab^ ± 3.99	85^b^ ± 3.09.
Milk (ml)	180^a^ ± 1.88	205^b^ ± 1.02	210^bc^ ± 0.99	215^c^ ± 0.90
*Moi-moi* (g)	170^b^ ± 2.68	192^b^ ± 2.01	215^c^ ± 1.97	210^c^ ± 1.08
Yoghurt (ml)	180^a^ ± 2.90	180^a^ ± 2.86	190^b^ ± 1.82	190^b^ ± 1.77
^c^Rice & beans jollof+vegetables (g)	210^a^ ± 2.99	282^b^ ± 2.12	340^c^ ± 2.01	348^c^ ± 1.99
Abatete C/SA PS (*N* = 206)	*N* = 84	*N* = 75	*N* = 24	N = 23
*Pap* + *richoco* (g)	170^a^ ± 1.72	200^b^ ± 0.87	220^c^ ± 0.07	220^c^ ± 0.04
*Okpa* (g)	180^a^ ± 0.78	220^b^ ± 0.88	220^b^ ± 0.81	220^b^ ± 0.01
Bread (g)	70^a^ ± 1.88	70^a^ ± 0.79	80^b^ ± 0.11	80^b^ ± 0.16
Egg (g)	70^a^ ± 1.43	70^a^ ± 1.43	70^a^ ± 1.43	70^a^ ± 1.43
Milk (ml)	180^a^ ± 0.57	210^b^ ± 0.88	220^c^ ± 0.88	240^d^ ± 0.02
*Moi-moi* (g)	170^a^ ± 2.36	200^b^ ± 0.92	220^c^ ± 0.51	220^c^ ± 0.19
Soya bean milk (ml)	180^a^ ± 0.91	210^b^ ± 0.92	210^b^ ± 0.42	240^c^ ± 0.18
^c^Rice & beans jollof+vegetables (g)	260^a^ ± 0.06	340^b^ ± 0.45	420^c^ ± 0.24	440^d^ ± 0.11

*Abatete C/SA PS* Abatete Central/Salvation Army Primary School, *Oma-Eke CPS* Oma-Eke Community Primary School, *Richoco* Cocoa powder from Cadbury, *Okpa* Steamed Bambara groundnut pudding, *Moi-moi* Steamed cowpea pudding, *Pap* Maize paste prepared in hot water

^c^This was consumed with a finger of banana

Values with different superscripts in the same row are significantly *(P < 0.05)* different

Values are means ± standard deviations

### Nutrient compositions of the school meals

The nutrient compositions of the meals consumed in the 3 schools are shown in Table 2. Egg had the highest value of protein (12.6 g), fat (9.5 g) and iron (1.7 g). Bread had the highest carbohydrate (48.4 g) and energy (243.4 Kcal) values. The highest vitamin A value was found in *okpa* (steamed bambara groundnut pudding) consumed at Eke (255.5 RE) while the highest vitamin C (8.2 mg) was found in steamed cowpea pudding *(moi-moi)* consumed at Oma-Eke. Yogurt (118.0 mg) and steamed bambara groundnut pudding (60.0 mg) were superior to other foods in calcium. Rice and beans consumed at Oma-Eke proved superior in zinc to other foods consumed in all the schools.

**Table 2:** Nutrient composition of school meals consumed by the children in the three schools

Food	Protein (%)	Carbohydrate (%)	Fat (%)	Energy (Kcal)	Ash (%)	Moisture (%)	Fibre (%)	Vitamin A (RE)	Vitamin C (mg)	Iron (%)	Calcium (mg)	Zinc (mg)
Eke Central School												
^a^Yellow *pap*	2.8	28.4	0.3	127.5	0.1	67	1.3	3	NA	0.1	3	0.14
*Okpa*	9.6 ± 0.28	27.2 ± 0.22	4.70 ± 0.23	189.5	0.53 ± 0.06	56.42 ± 0.01	1.55 ± 0.21	255.5 ± 5.01	8.11 ± 0.01	0.65 ± 0.07	50 ± 0.01	1.50 ± 0.11
*Richoco*	0.69	8.2	0.19	37.27	NA	NA	0.1	187.5	4.5	1.35	18	1.13
Rice & beans	9.2 ± 0.47	20.5 ± 0.38	4 ± 0.09	145.6	1.2 ± 0.02	64.1 ± 0.03	3.34 ± 0.42	159.6 ± 9.61	6.7 ± 0.00	0.5 ± 0.00	45 ± 0.01	1.3 ± 0.10
^a^Bread	8.4	48.4	1.8	243.4	1.9	36.5	3.1	63.2	NA	1.2	28	0.6
^a^Egg	12.6	0.7	9.5	138.7	1.1	76.2	NA	152	NA	1.7	53	1.23
^a^Milk	3.5	4.8	1.6	47.6	1	89.3	NA	33	1.5	0.05	120	0.39
*Moi-moi*	5.3 ± 0.27	15.37 ± 0.37	2.97 ± 0.18	109.41	0.93 ± 0.09	72.18 ± 0.09	1.1 ± 0.49	97.8 ± 7.71	4.62 ± 0.93	0.38 ± 0.06	50 ± 0.00	1.12 ± 0.09
^a^Yoghurt	3.8	6.8	3.4	73	0.8	85.3	NA	30	0.7	0.1	118	0.58
Oma-Eke Community Primary School												
^a^Yellow *pap*	2.8	28.4	0.3	127.5	0.1	67	1.3	3	NA	0.1	3	0.14
*Okpa*	9.7 ± 0.28	28.1 ± 0.22	4.96 ± 0.23	195.8	1.02 ± 0.06	55.52 ± 0.15	0.7 ± 0.21	70.5 ± 5.01	6.73 ± 0.00	0.27 ± 0.07	42 ± 0.00	0.74 ± 0.02
*Richoco*	0.69	8.2	0.19	37.27	NA	NA	0.1	187.5	4.5	1.35	18	1.13
Rice & beans	7.2 ± 0.47	20.1 ± 0.28	3.6 ± 0.09	141.4	1.4 ± 0.08	66.2 ± 0.47	1.6 ± 0.14	129.7 ± 5.89	7.0 ± 0.00	0.3 ± 0.04	30 ± 0.04	3.0 ± 0.13
^a^Bread	8.4	48.4	1.8	243.4	1.9	36.5	3.1	NA	NA	1.2	28	0.6
^a^Egg	12.6	0.7	9.5	138.7	1.1	76.2	NA	152	NA	1.7	53	1.23
^a^Milk	3.5	4.8	1.6	47.6	1	89.3	NA	33	1.5	0.05	120	0.39
*Moi-moi*	8.42 ± 0.49	14.91 ± 0.01	3.35 ± 0.19	128.87	1.74 ± 0.10	70.28 ± 0.42	1.30 ± 0.14	135.6 ± 4.45	8.22 ± 0.00	0.79 ± 0.73	40 ± 0.00	1.21 ± 0.04
^a^Yoghurt	3.8	6.8	3.4	73.0	0.8	85.3	NA	30	0.7	0.1	118	0.58
Abatete Central/Salvation Army Primary School												
^a^Yellow *pap*	2.8	28.4	0.3	127.5	0.1	67	1.3	3	NA	0.1	3	0.14
*Okpa*	7.31 ± 0.22	15.6 ± 0.50	5.02 ± 0.06	134.6	2.53 ± 0.13	68.59 ± 0.23	0.95 ± 0.24	174.9 ± 9.80	5.25 ± 0.00	0.81 ± 0.09	60 ± 0.00	0.67 ± 0.07
*Richoco*	0.69	8.2	0.19	37.27	NA	NA	0.1	187.5	4.5	1.35	18	1.13
*Moi-moi*	7.8 ± 0.30	15.2 ± 0.37	3.0 ± 0.18	119.3	1.0 ± 0.09	71.1 ± 0.46	2.0 ± 0.35	93.8 ± 7.71	7.3 ± 0.00	0.4 ± 0.04	40 ± 0.01	0.8 ± 0.06
^a^Bread	8.4	48.4	1.8	243.4	1.9	36.5	3.1	NA	NA	1.2	28	0.6
^a^Egg	12.6	0.7	9.5	138.7	1.1	76.2	NA	152	NA	1.7	53	1.23
^a^Milk	3.5	4.8	1.6	47.6	1	89.3	NA	33	1.5	0.05	120	0.39
Rice & beans	8.24 ± 0.49	12.90 ± 0.09	3.99 ± 0.04	120.4	0.72 ± 0.27	69.95 ± 0.16	4.20 ± 0.57	36.2 ± 0.71	5.02 ± 0.00	0.50 ± 0.13	40 ± 0.01	1.22 ± 0.04
^a^Soya bean milk	3.4	1	1.5	41.5	1	93	0.4	NA	NA	0.6	12	NA

*Moi-moi* Steamed cowpea pudding, *Pap* Maize paste prepared in hot water, *Richoco* Cocoa powder from Cadbury, *NA* Not Available

^a^Values were obtained from West African food Composition Table (2012) CHO = carbohydrate Calc = calcium *Okpa* = Steamed Bambara groundnut pudding

### Energy and nutrient intakes of the school children

The energy and nutrient intakes of the school children and the percentage of energy from the energy giving nutrients are shown in Table 3. In all the schools, the intakes increased with age with the 2 – 5-year-olds having the least (*P < 0.05*) intakes. Carbohydrate was the main source of energy from the school meals with protein and fat making similar contributions to energy intake. Protein intake ranged from 18.11 - 27.27 g and carbohydrate ranged from 50.28 – 71.31 g. Fat and energy intakes ranged from 8.78 – 14.86 g and from 357.01 – 513.72 kcal, respectively. Vitamin A intake ranged from 214.75 – 353.75 RE while vitamin C ranged from 11.45 – 17.87 mg. Calcium intake had a range of 132.90 – 215.81 mg; iron was 1.41 – 2.16 mg and the intake of zinc ranged from 2.10 to 5.90 mg.

**Table 3:** Nutrient and energy intakes of the school children and percentage of energy from the energy giving nutrients

Schools/Age groups	Protein (g) (% energy)	Carbohydrate (g) (% energy)	Fat (g) (% energy)	Energy (Kcal)	Vitamin A (mg)	Vitamin C (mg)	Calcium (mg)	Iron (mg)	Zinc (mg)
6 – 9 years	23.47^b^(22.4)	58.18^ab^(55.5)	10.32^b^(22.1)	410.58^b^	300.78^b^	13.07^ab^	193.79^b^	1.88^a^	3.57^a^
10 – 14 years	26.80^c^(22.8)	64.42^bc^(54.8)	11.69^c^(22.4)	439.24^c^	330.71^c^	15.12^b^	212.61^c^	2.08^b^	4.09^b^
15 – 18 years	27.10^c^(22.7)	65.39^c^(54.9)	11.86^c^(22.4)	466.91^d^	335.82^c^	15.23^b^	215.81^c^	2.09^b^	4.11^b^
Oma-Eke Community Primary School									
2 – 5 years	18.81^a^(20.4)	52.23^a^(56.8)	9.32^a^(22.8)	369.43^a^	261.48^a^	12.64^a^	154.70^a^	1.41^a^	4.23^a^
6 – 9 years	22.90^b^(20.3)	64.60^b^(57.3)	11.24^ab^(22.4)	452.80^b^	318.98^b^	16.01^b^	176.84^b^	1.62^b^	5.20^b^
10 – 14 years	25.10^c^(20.3)	71.09^c^(57.4)	12.31^bc^(22.3)	497.42^c^	338.74^bc^	17.79^b^	188.99^c^	1.73^c^	5.90^b^
15 – 18 years	25.10^c^(20.2)	71.31^c^(57.3)	12.47^bc^(22.5)	497.87^c^	353.75^c^	17.68^b^	190.60^c^	1.72^c^	5.87^b^
Abatete Community Primary School									
2 – 5 years	18.11^a^(20.1)	52.09^a^(57.9)	8.78^a^(22.0)	359.82^a^	214.75^a^	13.50^a^	132.90^a^	1.74^a^	2.10^a^
6 – 9 years	22.33^b^(20.6)	62.31^b^(57.4)	10.62^ab^(22.0)	434.14^b^	254.84^b^	15.28^a^	159.98^b^	2.08^b^	2.54^b^
10 – 14 years	24.43^ab^(20.7)	67.85^bc^(57.4)	11.48^ab^(21.0)	472.18^c^	265.70^c^	17.87^b^	171.18^c^	2.14^b^	2.79^b^
15 – 18 years	27.27^c^(20.8)	70.15^c^(53.6)	14.86^b^(25.6)	513.72^d^	264.16^c^	17.12^b^	172.24^c^	2.16^b^	3.02^c^

Values with different superscripts in the same column for each school are significantly *(P < 0.05)* different

### Percentage contributions of school meals to RNI

The contributions made by the school meals to the recommended nutrient and energy intakes of the school children in the 3 schools are presented in Table 4. The percentage of recommended energy and nutrient intakes met by the school meals consumed at Eke Central Primary School were protein (57.7 – 116.9%), energy (16.4 – 23.0%), calcium (16.3 – 28.5%), iron (7.2 – 20.6%), zinc (47.5 – 69.6%), vitamins A (82.7 – 150.4%) and C (37.3 – 38.2%). At Oma-Eke Community Primary School, the school meals provided 17.5 – 23.9% energy, 53.4 – 107.0% protein, 84.7 – 159.5% vitamin A, 67.6 – 92.9% zinc, 14.5 – 25.3% calcium, 5.9 – 15.7% iron and 42.0 – 45.7% vitamin C. The contributions of the school meals at Abatete were energy (18 – 24.3%), protein (58 – 103.1%), vitamin A (66.0 – 127.4), vitamin C (42.8 – 45.1%), zinc (35.1 – 45.4%), calcium (13.2 – 22.8%) and iron (7.4 – 19.3%).

**Table 4:** Percentage contribution of school meals to RNI of the school children in the three schools

Variables	Age groups	Energy (Kcal)	Protein (g)	Vitamin A (RE)	Vitamin C (mg)	Calcium (mg)	Iron (mg)	Zinc (mg)
Eke Central School								
Mean daily intake	2 – 5 years	357.01	20.45	266.18	11.45	171.15	1.85	3.34
FAO/WHO/UNU Requirement		1550	17.5	200	30	600	9	4.8
% contribution		23.0	116.9	133.1	38.2	28.5	20.6	69.6
Mean daily intake	6- 9 years	410.58	23.47	300.78	13.07	193.79	1.88	3.57
FAO/WHO/UNU Requirement		1950	27	200	35	700	16	5.6
% contribution		21.1	86.9	150.4	37.3	27.6	11.8	63.8
Mean daily intake	10 – 14 years	439.24	26.80	330.71	15.12	212.61	2.08	4.09
FAO/WHO/UNU Requirement	M	2200	34	400	40	1300	16	8.6
% contribution		19.9	78.8	82.7	37.8	16.3	13.0	47.5
FAO/WHO/UNU Requirement	F	1950	36	400	40	1300	16	7.2
% contribution		22.5	74.4	82.7	37.8	16.3	13.0	56.8
Mean daily intake	15 – 18 years	466.09	27.10	335.82	15.23	215.81	2.09	4.11
FAO/WHO/UNU Requirement	M	2850	47	400	40	1300	15	8.6
% contribution		16.4	57.7	83.9	38.1	16.6	13.9	47.8
FAO/WHO/UNU Requirement	F	2150	47	400	40	1300	29	7.2
% contribution		21.7	57.7	83.9	38.1	16.6	7.2	57.1
Oma-Eke Community Primary School								
Mean daily intake	2 – 5 years	369.43	18.81	261.48	12.64	154.70	1.41	4.23
FAO/WHO/UNU Requirement		1550	17.50	200	30	600	9	4.8
% contribution		23.8	107.0	130.1	42.0	25.2	15.7	88.0
Mean daily intake	6 – 9 years	465.42	22.90	318.98	16.01	176.84	1.62	5.20
FAO/WHO/UNU Requirement		1950	27	200	35	700	16	5.6
% contribution		23.9	84.8	159.5	45.7	25.3	10.1	92.9
Mean daily intake	10 – 14 years	497.42	25.10	338.74	17.79	188.99	1.73	5.90
FAO/WHO/UNU Requirement	M	2200	34	400	40	1300	16	8.6
% contribution		22.6	73.8	84.7	44.5	14.5	10.8	68.6
FAO/WHO/UNU Requirement	F	1950	36	400	40	1300	16	7.2
% contribution		25.5	69.7	84.7	44.5	14.5	10.8	81.9
Mean daily intake	15 – 18 years	497.87	25.10	353.75	17.68	190.60	1.72	5.82
FAO/WHO/UNU Requirement	M	2850	47	400	40	1300	15	8.6
% contribution		17.5	53.4	88.4	44.2	14.6	11.5	67.6
FAO/WHO/UNU Requirement	F	2150	47	400	40	1300	29	8.6
% contribution		23.0	53.4	88.4	44.2	14.6	5.9	67.6
Abatete Central/Salvation Primary School								
Mean daily intake	2 – 5 years	359.82	18.11	222.75	13.54	132.90	1.74	2.10
FAO/WHO/UNU Requirement		1550	17.5	200	30	600	9	4.8
% contribution		23.2	103.1	111.3	45.1	22.2	19.3	43.8
Mean daily intake	6 – 9 years	434.14	22.33	254.84	15.28	159.98	2.08	2.54
FAO/WHO/UNU Requirement		1950	27	200	35	700	16	5.6
% contribution		22.3	82.7	127.4	43.7	22.8	13.0	45.4
Mean daily intake	10 – 14 years	473.18	24.43	265.70	17.87	171.18	2.14	2.79
FAO/WHO/UNU Requirement	M	2200	34	400	40	1300	16	8.6
% contribution		21.5	71.9	66.4	44.6	13.2	13.4	38.8
FAO/WHO/UNU Requirement	F	1950	36	400	40	1300	16	7.2
% contribution		24.3	67.9	66.4	44.6	13.2	13.4	38.8
Mean daily intake	15 – 18 years	513.72	27.27	264.16	17.12	172.24	2.16	3.02
FAO/WHO/UNU Requirement	M	2850	47	400	40	1300	15	8.6
% contribution		18.0	58.0	66.0	42.8	13.3	14.4	35.1
FAO/WHO/UNU Requirement	F	2150	47	400	40	1300	29	7.2
% contribution		23.9	58.0	66.0	42.8	13.3	7.4	41.9

Source: Energy and protein requirements: Report of a joint FAO/WHO/UNU expert consultation. Technical report series (724).WHO, Geneva,(1985)

Iron requirement: FAO report no 23 (1988). Requirements of vitamin A, calcium, iron and zinc, FAO/WHO report no 23 (1998)

## Discussion

This study aimed to assess the energy and nutrient composition of school meals served in 3 schools in Enugu and Anambra States of Nigeria and their contributions to energy and nutrient intakes of school children. It showed that pulses were consumed on 4 days out of five school days in a week. Cowpeas and bambara groundnuts are among the pulses recognized by Food and Agriculture Organization (FAO) [19] because of their contributions to protein and micronutrient intakes of individuals. Pulses typically contain about twice the amount of protein found in whole grain cereals and are rich sources of vitamins, iron, zinc, magnesium, bioactive compounds and gives a better protein quality when consumed with cereals [19, 20].

### Portion sizes of the school meals consumed by the pupils

In all the 3 schools, the portion sizes consumed by the 2 – 5-year olds were the smallest. This was expected because they cannot consume a quantity that is beyond their gastric capacity. A study showed that the intake of young children remained the same irrespective of the portion size served [21]. This implies that large portion sizes are not necessary for these children rather the emphasis should be on nutrient dense portion sizes.

### Energy and nutrient composition of the school meals

The nutrient compositions of the foods per 100 g were in line with the results reported by other researchers [4, 22, 23] on similar foods. These nutrients build and maintain healthy tissues; aid body processes, protect the body against infections and diseases; and provide energy for work [24]. Several dietary components have been identified as having positive effects on cognitive abilities. Dietary factors such as iron, zinc, copper, vitamins B, D, E, C and carotenes can affect multiple brain processes by regulating neurotransmitter pathways, synaptic transmission, membrane fluidity and signal-transduction pathways [25]. This implies that school children can benefit physically and cognitively from school meals. This benefit spans from the nutrients in the meals and its ability to add to total energy and nutrient intakes.

### Energy and nutrient intakes of the school children

In all the schools, the intakes increased with age with the 2 – 5-year-olds having the lowest intakes. This is in line with the portion sizes consumed. It was not surprising that the energy and nutrient intakes of the children increased with age since the portion sizes had similar trend. Rolls et al. [21] showed that larger portion sizes lead to greater energy intake regardless of serving method. The energy expenditure of older children may be higher; therefore a larger portion size prevents negative energy balance and nutrition/health problems associated with it.

That carbohydrate was the main source of energy from the school meals with protein and fat making similar contributions to energy intake was expected. This is necessary to provide readily available energy for school work. Carbohydrates (sugars and starches) provide energy to cells in the body particularly the brain which is a glucose-dependent organ [26]. It also ensures that protein is spared for the function of growth, repair and maintenance of body tissues. The percentage of carbohydrate, fat and protein contributing to energy intake is in line with the dietary guidelines that 45 - 65% of energy be supplied by carbohydrate, 25 – 40% by fat and 10 – 35% by protein [26]. However, the value suggests the need to increase fat intake.

### Contribution of the school meals to energy and nutrient intakes of the pupils

School meals should provide at least one third (an equivalent of 33.3%) of the daily recommended nutrient intake (RNI) for energy, protein and other nutrients [27]. The foods consumed by school children in these schools provided more than one third of the RNI for protein, zinc, vitamins A and C but failed to meet a third of the RNI for calcium, iron and energy. These findings are in agreement with the outcome of other researches. Nelson et al. [28] showed that 4 – 18-year-old pupils in England who received free school meals derived a significantly greater proportion of their daily energy and nutrient intakes from their school meals than those who did not have a free school meal. School canteen lunches provided the most nutritious lunch for Scottish school children, with street lunches providing the least nutritious lunch [29]. A study by Owusu et al. [30] in Ghana also showed that the meals provided by Non-Governmental School Feeding Programme (NSFP) had larger portion sizes and contributed 28 and 24.6% to energy and protein intakes of the children, respectively. Other researchers [31–33] have reported similar findings.

The inadequate contribution made by the foods to energy, calcium and iron requirements of the school children in the 3 schools was in agreement with the report of Nelson et al. [28] who reported that school meals failed to make good the shortfalls in daily intakes of calcium, iron, zinc, and vitamin A. Our finding was attributed to the portion sizes given the pupils which were not nutrient dense enough to provide the required one third of the energy requirement. Besides, we found that only milk and yogurt provided over 100 mg calcium per 100 g suggesting that a larger quantity and frequency of consumption may increase calcium intake. Use of leafy vegetables alone was insufficient. Factors like cost and availability of funds may have affected the portion sizes due to changes in the school menu with the subsequent effect on the energy and nutrient intakes. Cummings et al. [34] affirmed that menu changes resulted in a net reduction of calories, sugar and sodium content of meals offered school children. It implies that other nutrients would be affected as well.

Results from studies like this are important for effective policy formulation and implementation especially where nutrition interventions are required. Previous reports [1, 34] have indicated essential roles of school meals in determining adequate health status and general development of school children especially those in areas where school health programme is poorly implemented and school lunch programme not implemented. The limitation of the study is that it focused only on the meals served in PACIEH’s schools since these were the only schools where school meal programme was implemented in the two states. Besides, we did not put into consideration the home meals consumed by the children and the bioavailability of the nutrients especially iron and zinc.

### Recommendation

PACIEH should review the school menu to ensure that the quantity of rich sources of calcium and iron are increased. The number of times milk/yogurt and eggs are consumed in a week should be increased. Energy and calcium intake can be increased by use of calcium fortified spreads for bread.

## Conclusions

The meals consumed by the school children in all the three schools where PACIEH implemented school feeding were nutrient dense and provided a third of the recommended daily intakes of protein, zinc, and vitamins A and C but failed to meet that of calcium, iron and energy. Calcium and iron are critical nutrients required by school children and calls for a review of the recipes and frequency of consumption of the meals. It is hoped that the results of this study will serve as a guide for future planning of school feeding programmes in Nigeria and more specifically the national Home-grown School Feeding (HGSF) programme being implemented in many States of the Federal Republic of Nigeria.

## Abbreviations

ENSUBEB: Enugu State Universal Basic Education Board
HAF: Heineken African Foundation
PACIEH: Pan African Community Initiative on Education and Health
PTA: Parents Teachers’ Association
RNI: Recommended nutrient intake
SBMC: School based management committee
